# Decellularized Adipose Matrix Rejuvenates Photoaged Skin through Immune Microenvironment Modulation

**DOI:** 10.34133/bmef.0166

**Published:** 2025-08-04

**Authors:** Jialiang Zhou, Shengjie Jiang, Liyun Wang, Kaili Lin, Jianyong Wu, Haijun Gui, Zhen Gao

**Affiliations:** ^1^ Department of Stomatology, Xin Hua Hospital, Shanghai Jiao Tong University School of Medicine, Shanghai 200092, China.; ^2^ Department of Oral and Cranio-Maxillofacial Surgery, Shanghai Ninth People’s Hospital, Shanghai Jiao Tong University School of Medicine; College of Stomatology, Shanghai Jiao Tong University; National Center for Stomatology; National Clinical Research Center for Oral Diseases, Shanghai Key Laboratory of Stomatology, Shanghai Research Institute of Stomatology, Shanghai 200011, China.; ^3^ Department of Plastic and Reconstructive Surgery, Shanghai Ninth People’s Hospital, Shanghai Jiao Tong University School of Medicine, Shanghai 200011, China.

## Abstract

**Objective:** This study aims to explore the therapeutic potential of decellularized adipose matrix (DAM) in rejuvenating photoaged skin by modulating the immune microenvironment. **Impact Statement:** DAM effectively induces M1 to M2 macrophage polarization and rescues the function of photoaged fibroblasts through paracrine mechanisms, providing a novel strategy for skin antiaging through immune microenvironment remodeling. **Introduction:** Photoaging, triggered by prolonged ultraviolet exposure, is marked by the depletion of skin structural elements and a persistent inflammatory environment. Current clinical interventions primarily target structural defects, while immune modulation remains underexplored. Therefore, developing biomaterials with both extracellular matrix (ECM) replenishment and immune regulatory functions is crucial for skin regeneration. **Methods:** A photoaged mouse model was established using ultraviolet B irradiation to validate the inflammatory microenvironment. DAM was prepared via physicochemical decellularization and assessed in vitro for its effects on macrophage polarization and macrophage-fibroblast cross-talk. A DAM-functionalized hyaluronic acid (HA/DAM) hydrogel was developed and evaluated for its effects on skin rejuvenation via subcutaneous injection. **Results:** In vitro experiments demonstrated that DAM substantially promoted M2 macrophage polarization, and M2-macrophage-conditioned medium further improved fibroblast functions, including oxidative stress resistance, migration, and ECM synthesis. In vivo, HA/DAM hydrogel not only increased dermal thickness and collagen density but also restructured the immune microenvironment through M2 macrophage polarization. **Conclusion:** DAM offers a novel therapeutic approach for skin rejuvenation by modulating the immune microenvironment, demonstrating notable clinical potential.

## Introduction

Photoaging is a chronic pathological process caused by prolonged exposure to ultraviolet radiation (UVR), characterized by wrinkles, skin laxity, roughness, and hyperpigmentation [1]. UVR induces cellular senescence and generates excessive reactive oxygen species (ROS), leading to cellular dysfunction and structural deterioration of tissues. In addition, it activates matrix metalloproteinases (MMPs), which degrade extracellular matrix (ECM), thus compromising skin integrity [2].

Globally, increased UVR exposure has markedly elevated the prevalence of photoaging. Over 83% of individuals aged 20 and above display prominent signs of photoaging [3], while 72% of men and 47% of women under 30 exhibit moderate to severe alterations in skin texture [4]. Beyond its cosmetic implications, photoaging is recognized as a precursor to more severe health risks, such as nonmelanoma skin cancer (NMSC). In 2019, approximately 28.4% of individuals of working age worldwide faced occupational UVR exposure, contributing to 18,960 NMSC-related deaths and 500,000 disability-adjusted life years. Notably, the disease burden of UVR-associated NMSC nearly doubled between 2000 and 2019 [5]. Given its widespread prevalence, associated health risks, and escalating disease burden, photoaging has emerged as a critical public health challenge.

The pathogenesis of photoaging is multifaceted, involving not only the loss of structural components but also the establishment of a chronic inflammatory microenvironment [6,7]. Alterations in the ECM modulate the trafficking and recruitment of immune cells through both physical properties and bioactive components, thereby shaping the local immune microenvironment. Conversely, chemokines and cytokines secreted by immune cells can remodel the ECM structure, establishing a dynamic feedback loop [8]. Among these, macrophages, which are pivotal regulators of skin tissue homeostasis, exhibit functional states intricately tied to the local microenvironment. Specifically, proinflammatory M1 macrophages have been shown to directly contribute to excessive degradation of the dermal ECM, while anti-inflammatory M2 macrophages are associated with the secretion of anti-inflammatory cytokines and tissue regeneration functions [9]. Current investigations have further underscored the essential regulatory role of macrophage functional states and their interactions with fibroblasts in photoaging mechanisms [10,11].

Within the photoaged dermis, macrophages are typically adjacent to senescent fibroblasts, which robustly drive macrophage polarization into the M1 state via the release of senescence-associated secretory phenotype [12]. Gather et al. [13] have demonstrated that the percentage of M1 macrophages in aging skin escalates by over 50%, and the proinflammatory cytokines they release substantially aggravate ECM metabolic dysfunction in fibroblasts. Moreover, in vitro coculture experiments reveal that interactions with senescent fibroblasts markedly enhance M1 polarization of macrophages, underscoring that the chronic inflammatory microenvironment dynamically formed by these cells is a key contributor to driving the pathological progression of photoaging.

Despite progress in elucidating the mechanisms behind photoaging, existing therapeutic approaches remain limited in addressing the underlying inflammatory microenvironment. Current clinical strategies for managing photoaging predominantly encompass dermal fillers, antioxidants, retinoids, and laser therapies [14]. These methods primarily focus on addressing structural deficiencies, such as collagen degradation, by enhancing collagen synthesis, suppressing MMP activity, or improving aesthetic outcomes. Recently, advanced biomaterials with immunomodulatory properties, including extracellular vesicles, nanomaterials, and functionalized hydrogels, have emerged as promising candidates [15–18]. Nevertheless, their translation into clinical practice is hindered by challenges such as ethical concerns and limited availability of donor resources. Consequently, the development of innovative therapies that concurrently rectify structural deficits and regulate the inflammatory microenvironment is of paramount importance for the effective management of photoaging.

In this context, the development of decellularized ECM (dECM) has opened new avenues for photoaging treatment. As a commonly used bioink, dECM delivers structural scaffolding and bioactive signaling molecules to actively promote tissue repair and regeneration [19,20]. Studies demonstrate that dECM regulates macrophage polarization through diverse mechanisms, exerting anti-inflammatory and prorepair effects. For instance, porcine-skeletal-muscle-derived dECM activates the phosphatidylinositol 3-kinase/protein kinase B (PI3K/Akt) pathway, thereby reducing iron deposition and promoting M2 polarization [21]. Similarly, freeze-thaw-treated porcine peritoneal dECM enhances M2 polarization via mechanotransduction-mediated nuclear factor κB (NF-κB) modulation [22], while decellularized amniotic membrane suppresses Toll-like receptor (TLR) and tumor necrosis factor (TNF) signaling to attenuate inflammation [23]. These findings collectively highlight dECM’s potential in photoaging treatment.

However, dECM encounters challenges, including limited donor availability, prompting the exploration of alternative sources such as decellularized adipose matrix (DAM). DAM has gained prominence as a promising candidate due to its abundant availability and low ethical barriers. Several DAM-based products have advanced to clinical use, demonstrating favorable biosafety and efficacy in preclinical and clinical studies [24,25]. On the basis of these findings, we hypothesize that DAM, by inducing M2 macrophage polarization, can effectively reprogram the inflammatory microenvironment and restore both structural integrity and functional homeostasis in photoaged skin.

To test this hypothesis, we first validated the inflammatory microenvironment in photoaged skin using a mouse model. DAM was derived from adipose tissue and was shown to induce M2 macrophage polarization in vitro. Further research revealed that conditioned medium (CM) from DAM-induced macrophages substantially improved the functional recovery of photoaged fibroblasts. Finally, DAM combined with hyaluronic acid (HA) hydrogel was administered to the photoaged mouse model, resulting in enhanced collagen synthesis through M2 macrophage polarization. These discoveries emphasize the potential of DAM as a viable therapeutic option for photoaging, providing a dual approach to modulate the inflammatory microenvironment and restore skin structure.

## Results and Discussion

### Ultraviolet B irradiation induces inflammatory microenvironment in skin photoaging

**Fig. 1.**
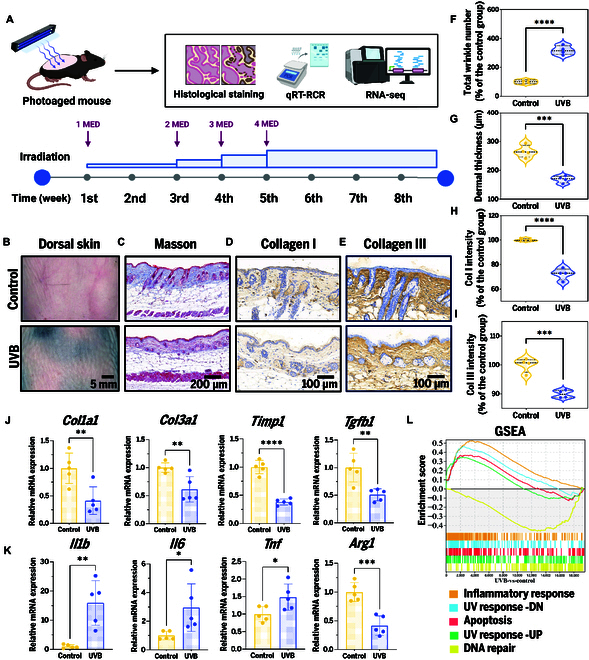
UVB irradiation induces inflammatory microenvironment in skin photoaging. (A) Modeling procedure and UVR intensity. (B) Macroscopic images. Scale bar, 5 mm. (C) Masson’s trichrome staining images. Scale bar, 200 μm. (D) IHC staining images of collagen I. Scale bar, 100 μm. (E) IHC staining images of collagen III. Scale bar, 100 μm. Statistical analysis of (F) total wrinkle number, (G) dermal thickness, (H) collagen I (col I) intensity, and (I) collagen III (col III) intensity. (J) qRT-PCR results related to ECM synthesis. (K) qRT-PCR results related to inflammation. *P < 0.05; **P < 0.01; ***P < 0.001; ****P < 0.0001. (L) GSEA. DN, down-regulated; UP, up-regulated.

Given the high structural and pathological similarity between mouse and human skin in photoaging [26], C57BL/6 mice were selected as the animal model for this study. To establish the photoaging model, we exposed mice to an ultraviolet B (UVB) gradient-enhanced irradiation protocol spanning 8 weeks [27] (Fig. 1A). Macroscopic examination revealed the formation of prominent deep wrinkles on the dorsal skin (Fig. 1B), with quantitative analysis confirming significant increases in wrinkle parameters (Fig. 1F), thereby confirming the successful induction of the phenotypes. Masson’s trichrome staining (Fig. 1C and G) showed a notable decrease in dermal thickness and disrupted collagen fiber organization, reflecting severe ECM structural damage. To further validate these findings, we performed immunohistochemical (IHC) staining, demonstrating substantial degradation of both collagen I (Fig. 1D and H) and collagen III (Fig. 1E and I) in photoaged skin tissues, thus providing additional evidence for the loss of key ECM components.

To explore collagen synthesis mechanisms in photoaging, we performed quantitative reverse transcription polymerase chain reaction (qRT-PCR) analysis. The results showed significant down-regulation of collagen type I α1 chain (*Col1a1*), collagen type III α1 chain (*Col3a1*), tissue inhibitor of metalloproteinases 1 (*Timp1*), and transforming growth factor-β1 (TGF-β1; *Tgfb1*), suggesting a marked suppression of collagen synthesis (Fig. 1J). Collectively, these findings demonstrate that the UVB-induced photoaging mouse model effectively recapitulates the critical pathological features of human skin photoaging.

Given the critical function of immune microenvironment in photoaging, we further analyzed the expression profiles of inflammation-related genes. Our findings demonstrated that UVB exposure markedly increased the levels of proinflammatory mediators—interleukin-1β (IL-1β; *Il1b*), IL-6 (*Il6*), and *Tnf*—while suppressing the expression of the anti-inflammatory mediator arginase 1 (*Arg1*), providing direct evidence for the formation of a proinflammatory state (Fig. 1K). To further validate the inflammatory microenvironment, we conducted the transcriptome sequencing on mouse skin tissues. Gene set enrichment analysis (GSEA) revealed significant enrichment of the “inflammatory response” pathway, further confirming its presence. Further analysis of the transcriptomic data showed that the “ultraviolet response” and “apoptosis” pathways were significantly up-regulated, whereas the “DNA repair” pathway was down-regulated, indicating that UVB irradiation induces extensive transcriptomic reprogramming (Fig. 1L).

The results presented above demonstrate that this study successfully established a mouse model of skin photoaging via UVB irradiation and uncovered the crucial involvement of the inflammatory microenvironment in advancing photoaging. These results provide critical experimental data to enhance insights into the pathological processes associated with photoaging, while also suggesting potential theoretical foundations for designing rejuvenation therapies targeting the immune microenvironment in photoaged skin.

### Preparation and characterization of DAM

**Fig. 2.**
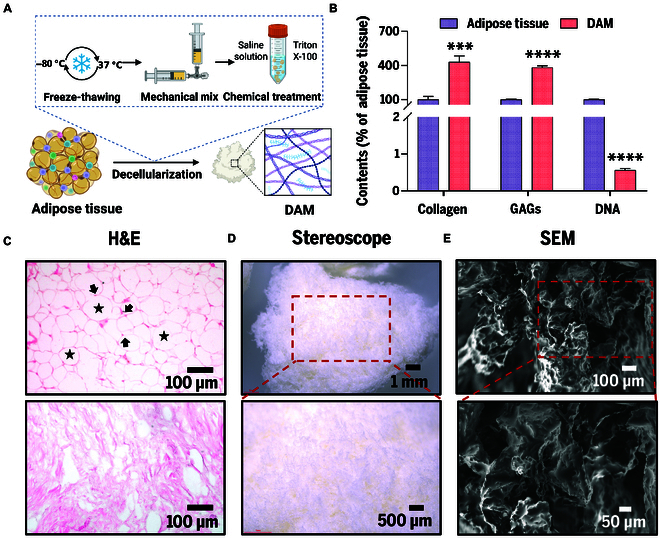
Preparation and characterization of DAM. (A) Illustration of preparation procedures. (B) Relative contents of collagen, GAGs, and DNA in DAM and original adipose tissues, with original adipose tissues set as 100% for comparison. ***P < 0.001; ****P < 0.0001. (C) H&E staining images (arrows indicate cell nuclei; stars denote lipid droplets). Scale bars, 100 μm. (D) Optical images. Scale bars, 1 mm (overview) and 500 μm (detailed view). (E) SEM images. Scale bars, 100 μm (overview) and 50 μm (detailed view).

To develop novel biomaterials with both immunomodulatory and ECM-mimetic functions, we extracted DAM using a combined physical–chemical decellularization strategy [28]. As shown in Fig. 2A, adipose tissue obtained through liposuction was subjected to 3 cycles of freezing and thawing (−80 °C/37 °C) for disrupting adipocyte membranes, followed by mechanical emulsification using a nanofat converter. Subsequently, cellular debris and residual lipids were removed sequentially with hypertonic saline and Triton X-100.

The decellularization efficiency was evaluated through biochemical quantification and histological analysis. DAM had less than 1% DNA content compared to adipose tissue, confirming efficient decellularization (Fig. 2B). Hematoxylin and eosin (H&E) staining further verified the decellularization efficacy: The densely packed cell nuclei and lipid droplet vacuoles observed in the native adipose tissue were absent in the DAM, yielding a loose ECM network, indicating a marked reduction in immunogenicity (Fig. 2C). Optical microscopy revealed that the freeze-dried DAM displayed a white, fluffy, and porous structure (Fig. 2D). Scanning electron microscopy (SEM) showed that the DAM retained the 3-dimensional fibrous network, closely resembling natural dermal ECM and supporting cell migration and signal transduction (Fig. 2E). In addition, the DAM largely preserved collagen (4.32-fold higher than equivalent freeze-dried adipose tissue) and glycosaminoglycans (GAGs) (3.79-fold higher than equivalent freeze-dried adipose tissue), demonstrating the effective retention of native ECM components (Fig. 2B).

These results demonstrate that the decellularization strategy adopted in this study integrates high efficiency with biocompatibility. In contrast to conventional enzymatic methods, the enzyme-free treatment avoids excessive degradation of ECM proteins while ensuring substantial elimination of residual adipocytes. This feature establishes a solid basis for directly replenishing ECM components in photoaged skin and facilitating structural repair.

### DAM reverses M1 macrophage polarization and promotes M2 polarization

Macrophages are essential for preserving the balance of the tissue microenvironment [29]. In photoaged skin, hyperactivated M1 macrophages drive chronic local inflammation through the secretion of inflammatory mediators, including IL-1β, IL-6, and TNF-α, thereby accelerating skin aging [30]. In contrast, M2 macrophages are closely linked to tissue regeneration and repair, and their secretion of anti-inflammatory factors, such as IL-4, IL-10, and TGF-β, promotes inflammation resolution and tissue healing [31]. Given previous evidence that dECM can induce M2 macrophage polarization, we hypothesized that DAM might exhibit similar regulatory properties and further explored its effects on macrophage polarization.

**Fig. 3.**
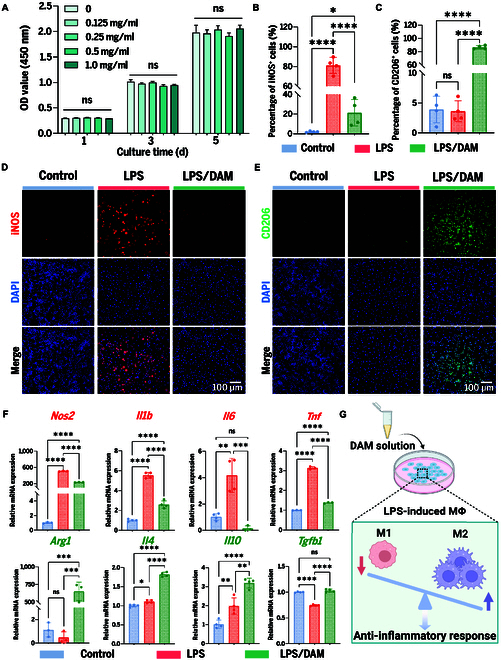
In vitro evaluation of DAM-mediated inflammatory modulation. (A) Proliferation of RAW264.7 cells treated with DAM. OD, optical density. (B) Analysis of iNOS-positive cells. (C) Analysis of CD206-positive cells. *P < 0.05; ****P < 0.0001. (D) Immunofluorescence images of iNOS staining. Scale bar, 100 μm. (E) Immunofluorescence images of CD206 staining. Scale bar, 100 μm. (F) qRT-PCR analysis of proinflammatory and anti-inflammatory cytokine mRNA levels. *P < 0.05; **P < 0.01; ***P < 0.001; ****P < 0.0001. (G) Illustration of the mechanism of DAM-mediated inflammatory modulation. MΦ, macrophage.

In vitro, DAM was initially dissolved through pepsin-mediated enzymatic digestion and then formulated as a concentrated solution of 10 mg/ml. To assess the biosafety of DAM, we tested its impact on macrophage proliferation with the Cell Counting Kit-8 (CCK-8) assay. The findings indicated that DAM, at doses ranging from 0.125 to 1.0 mg/ml, showed no notable suppression of macrophage proliferation (Fig. 3A), thereby confirming its excellent biocompatibility. On the basis of these observations, a DAM dose of 0.5 mg/ml was selected for subsequent experimental studies.

Subsequently, the regulatory effects of DAM on macrophage polarization were assessed through immunofluorescence staining coupled with quantitative analysis. Following lipopolysaccharide (LPS) stimulation, the inducible nitric oxide synthase (iNOS)-positive rate in RAW264.7 cells significantly increased to 81.12 ± 9.12%, reflecting robust M1 polarization (Fig. 3B and D). In contrast, treatment with DAM for 3 d markedly reduced the iNOS-positive rate to 21.35 ± 13.38%, suggesting a suppression of M1 polarization. Concurrently, analysis of CD206 expression revealed that the CD206-positive rates in both the control and LPS groups were below 5%, whereas DAM treatment significantly elevated this rate to 86.67 ± 2.58% (Fig. 3C and E). These findings collectively demonstrate that DAM effectively counteracts M1 polarization and promotes a phenotypic transition toward the M2 state, highlighting its potential as a modulator of macrophage function.

To further substantiate these findings, we performed qRT-PCR analysis (Fig. 3F). Upon LPS stimulation, a significant up-regulation was observed in the gene expression of inflammatory factors, including nitric oxide synthase 2 (*Nos2*), *Il1b*, *Il6*, and *Tnf*. Conversely, DAM treatment elicited a marked increase in the levels of anti-inflammatory mediators, including *Arg1*, *Il4*, *Il10*, and *Tgfb1*. These findings collectively demonstrate that DAM effectively counteracts LPS-induced M1 polarization of macrophages and drives their phenotypic transition toward the M2 state (Fig. 3G).

The mechanism behind DAM-induced macrophage transition to the M2 phenotype may depend on the inherent ECM components of DAM and the regulation of essential signaling pathways. For instance, collagen, as a major ECM constituent, has been shown to bind to integrins such as α_1_β_1_ and α_2_β_1_, activating focal adhesion kinase, extracellular-signal-regulated kinase, and NF-κB pathways, which are thought to modulate inflammatory responses and promote M2 polarization [32,33]. Similarly, high-molecular-weight HA, a GAG abundant in DAM with reported anti-inflammatory properties, is proposed to bind to TLRs and CD44 receptors expressed on both innate and adaptive immune cells. This interaction appears to trigger signaling pathways that inhibit dendritic cell maturation and potentially enhance macrophage polarization toward the M2 phenotype, thereby contributing to anti-inflammatory effects [34]. Moreover, DAM is enriched with immunomodulatory proteins such as basic fibroblast growth factor and IL-10, which have been suggested to suppress proinflammatory pathways such as TLR/NF-κB while activating prorepair pathways such as PI3K/Akt, potentially enhancing the anti-inflammatory and tissue-regenerative functions of M2 macrophages [35]. Together, these findings suggest that DAM may utilize its bioactive components and supportive microenvironment to provide essential signals that drive macrophage polarization toward the M2 phenotype, although further mechanistic studies are needed to fully elucidate these processes.

### DAM enhances fibroblast function via macrophage CM

The skin is a multifaceted organ consisting of various cell types that collectively maintain local microenvironmental homeostasis through dynamic intercellular cross-talk. In this study, to investigate whether DAM-mediated modulation of macrophages could indirectly influence the functional state of dermal fibroblasts, the key cellular components responsible for ECM production, via paracrine signaling, we collected CM from macrophages under different treatment conditions and applied it to fibroblast cultures. This approach was used to elucidate the mechanisms by which DAM orchestrates the regulation of the skin microenvironment.

**Fig. 4.**
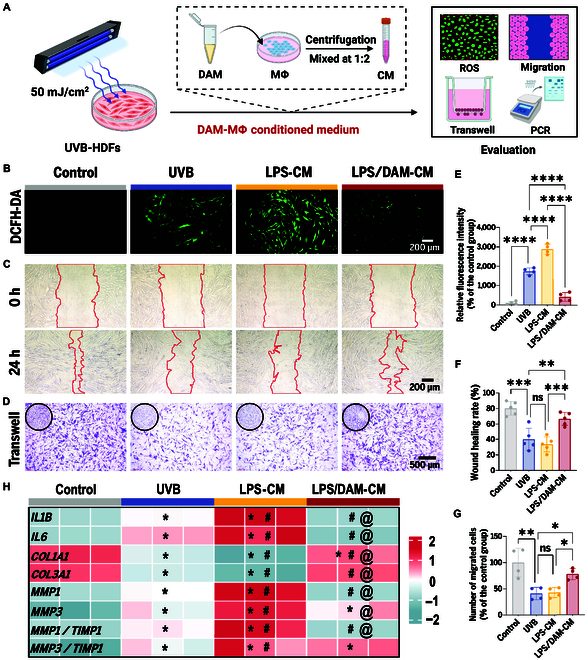
DAM enhances fibroblast function via macrophage CM. (A) Diagram depicting the photoaging HDF model, preparation of CM, and experimental evaluation. (B) Fluorescence images of intracellular ROS. Scale bar, 200 μm. (C) Images of wound healing assays. Scale bar, 200 μm. (D) Images of Transwell migration assays. Scale bar, 500 μm. (E) Quantification of relative ROS intensity. (F) Quantification of wound healing rates. (G) Quantification of relative migrated cells. *P < 0.05; **P < 0.01; ***P < 0.001; ****P < 0.0001. (H) qRT-PCR analysis of inflammation and ECM metabolism-related gene expression. *P < 0.05 versus control; #*P* < 0.05 versus UVB; @*P* < 0.05 versus LPS-CM (one-way ANOVA with Tukey’s post hoc test).

Human dermal fibroblasts (HDFs) were isolated from foreskin tissue using established protocols [36], and cells from passages 4 to 7 were used in all following studies. Given the central role of fibroblasts in maintaining skin structure and function, we focused on their response to environmental stressors. To recapitulate the pathological hallmarks of photoaging, we exposed HDFs to UVB irradiation and subsequently treated them with macrophage CM. The experimental design consisted of 4 groups: (a) the Control group, in which untreated HDFs were cultured with control-CM; (b) the UVB group, where HDFs subjected to UVB irradiation were cultured with control-CM; (c) the LPS-CM group, in which HDFs were cultured with CM from LPS-treated macrophages; and (d) the LPS/DAM-CM group, where HDFs were cultured with CM from macrophages cotreated with LPS and DAM (Fig. 4A).

Intracellular ROS generation was measured using the 2′,7′-dichlorodihydrofluorescein diacetate (DCFH-DA) probe (Fig. 4B and E). UVB irradiation markedly increased intracellular ROS levels, and this effect was further intensified by LPS-CM treatment. Importantly, LPS/DAM-CM treatment effectively reduced excessive ROS accumulation. Considering the importance of fibroblast migration in skin wound healing and tissue regeneration, the functional capacity was evaluated through scratch wound healing assays (Fig. 4C and F) and Transwell migration assays (Fig. 4D and G). UVB irradiation significantly inhibited fibroblast migration, whereas LPS-CM treatment did not exacerbate this inhibition. In contrast, LPS/DAM-CM treatment notably enhanced fibroblast migration. These results indicate that DAM modulates macrophage polarization to improve the inflammatory microenvironment, thereby promoting fibroblast migration and facilitating skin regeneration and repair.

qRT-PCR analysis (Fig. 4H) demonstrated that UVB irradiation significantly enhanced the transcriptional levels of proinflammatory cytokines (*IL1B* and *IL6*), and this effect was further intensified by LPS-CM treatment. However, LPS/DAM-CM treatment effectively reversed this trend. Moreover, UVB irradiation caused a significant reduction in the expression of key ECM components, *COL1A1* and *COL3A1*, which was further aggravated by LPS-CM treatment. In contrast, LPS/DAM-CM treatment significantly restored the expression of these genes. Furthermore, the ratio of *MMP1* and *MMP3* to their inhibitor (*TIMP1*) was notably elevated in both the UVB and LPS-CM groups, whereas this ratio was substantially reversed in the LPS/DAM-CM group. These findings indicate that the ECM metabolic balance in photoaged fibroblasts was disrupted and LPS-CM treatment exacerbated this pathological alteration. Conversely, LPS/DAM-CM treatment effectively restored ECM metabolic homeostasis, likely mediated by anti-inflammatory factors and prorepair signals secreted by M2-polarized macrophages.

In summary, the above results clearly demonstrate that DAM modulates the functional state of dermal fibroblasts indirectly by regulating macrophage polarization and altering their secretory cytokine profile.

### DAM-functionalized hydrogel improves photoaged skin via immune remodeling

**Fig. 5.**
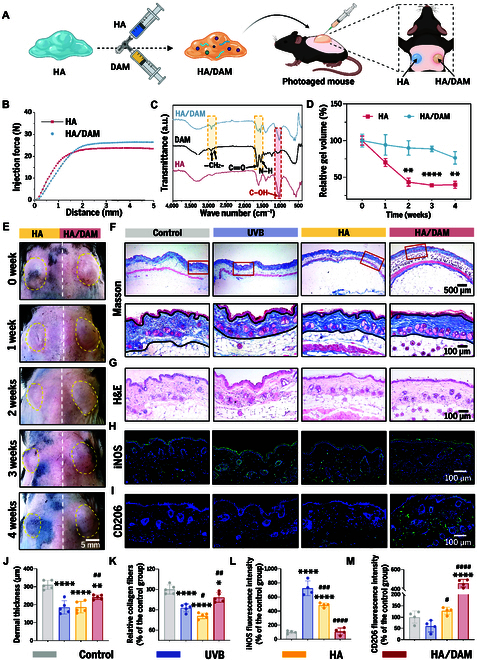
Therapeutic effects of DAM-functionalized hydrogel in photoaging mouse models. (A) Illustration of DAM-functionalized hydrogel preparation and injection. (B) Evaluation of injection force. (C) FTIR spectra of HA, DAM, and HA/DAM. a.u., arbitrary units. (D) Quantitative comparison of in vivo degradation kinetics between HA and HA/DAM hydrogels. **P < 0.01; ****P < 0.0001. (E) Photographs of injection regions. Scale bar, 5 mm. (F) Masson’s trichrome staining images. Scale bars, 500 μm (overview) and 100 μm (detailed view). (G) H&E staining images. Scale bar, 100 μm. (H) Immunofluorescence staining images of iNOS. Scale bar, 100 μm. (I) Immunofluorescence staining images of CD206. Scale bar, 100 μm. (J) Quantification of dermal thickness. (K) Quantification of relative collagen fibers density. (L) Quantification of iNOS signal intensity. (M) Quantification of CD206 signal intensity. In quantitative analyses, * and # denote significance versus the control and UVB groups, respectively. * and #, *P* < 0.05; ** and ##, *P* < 0.01; ###, *P* < 0.001; **** or ####, *P* < 0.0001.

In this study, we utilized commercially available HA hydrogel as a representative dermal filler to develop a novel therapeutic strategy for photoaging. DAM was physically encapsulated within the HA hydrogel using a syringe and a 3-way connector to fabricate HA/DAM hydrogel, and its therapeutic efficacy was assessed in a photoaged mouse model. A paired experimental design was used to compare the effects of HA hydrogel (HA group) and HA/DAM hydrogel (HA/DAM group), with HA hydrogel and HA/DAM hydrogel administered subcutaneously into the left and right back areas of the mice, respectively (Fig. 5A). In addition, the dermal microenvironment of the HA group and HA/DAM group was compared with that of normal mice (control group) and untreated photoaged mice (UVB group) to further validate the antiphotoaging effects of the functionalized hydrogel.

Injectability analysis (Fig. 5B) demonstrated that the incorporation of DAM moderately increased the injection force of the HA/DAM hydrogel, a phenomenon likely driven by the enhanced viscosity and flow resistance imparted by the DAM component. Notably, the maximum injection force for both hydrogels, measured using a 27-gauge needle, remained well below 30 N, fully compliant with the International Organization for Standardization 11040-4 standard for clinical applications. This ensures not only their safety but also their practical utility in clinical settings. Fourier transform infrared (FTIR) analysis (Fig. 5C) verified the coexistence of HA and DAM in the composite through their characteristic vibrational signatures. The spectrum clearly retained characteristic DAM signatures, including –CH_2−_ stretching (2,850 and 2,925 cm^−1^), amide I at 1,613 cm^−1^ (C═O stretching), and amide II at 1,560 cm^−1^ (N–H bending), alongside prominent HA-specific bands such as the C–OH stretching at 1,042 cm^−1^. Crucially, no new absorption bands were observed, confirming the structural integrity of both components. These findings collectively support a physical blending mechanism without covalent interactions between HA and DAM.

Maintaining hydrogel volume stability is crucial for achieving sustained antiwrinkle efficacy. Over a 4-week in vivo evaluation, the pure HA hydrogel exhibited markedly faster degradation compared to the HA/DAM hydrogel (Fig. 5D and E). Quantitative analysis revealed no significant difference in residual gel volume between HA and HA/DAM groups at week 1. However, from week 2 onward, the HA/DAM group demonstrated significantly greater volume retention compared to HA (Fig. 5D). This discrepancy can be attributed to the abundant collagen and GAG content within DAM, which synergistically enhances water retention and confers resistance to enzymatic degradation, thereby sustaining hydrogel volume over extended periods.

**Fig. 6.**
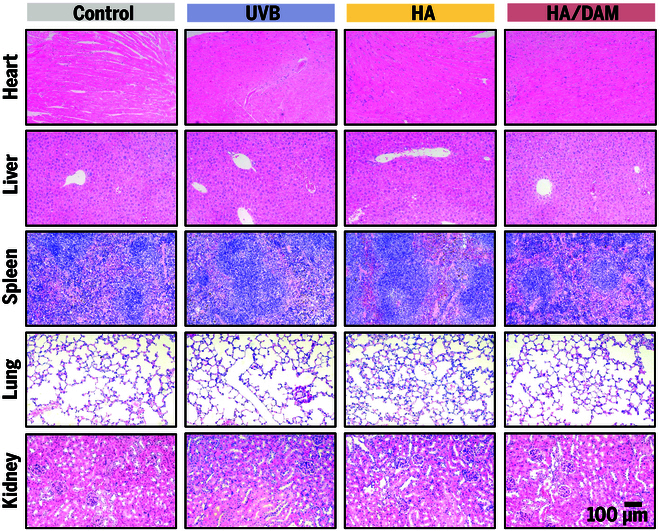
Representative H&E staining images of major organs in different groups. Scale bar, 100 μm.

At 4 weeks posttreatment, histological analysis of dorsal skin tissues in mice further underscored the superior performance of HA/DAM hydrogel. Masson’s trichrome staining revealed a marked increase in dermal thickness, accompanied by elevated collagen density and a highly organized, tightly packed collagen matrix in the HA/DAM group, indicative of enhanced tissue regeneration. In contrast, the HA group exhibited no significant improvement in dermal thickness (Fig. 5F, J, and K). H&E staining demonstrated that all experimental groups maintained intact skin tissue architecture, thereby confirming the excellent biocompatibility of the tested materials (Fig. 5G). To assess systemic safety, we performed H&E staining on major organs (heart, liver, spleen, lungs, and kidneys) from all groups (Fig. 6). All treated animals showed normal tissue architecture with no evidence of pathological changes, confirming the absence of systemic toxicity and supporting DAM’s potential for clinical translation.

Given the demonstrated potential of DAM to induce M2 macrophage polarization in vitro, we further explored its effects on inflammation regulation and immune microenvironment remodeling in vivo. Immunofluorescence staining for iNOS revealed a pronounced inflammatory response in the UVB group, with a significantly higher fluorescence signal intensity compared to the control group. Although the HA group showed a statistically significant reduction in fluorescence intensity compared to the UVB group, the reduction was less marked than in the HA/DAM group, which exhibited the most substantial decrease. These findings suggest that DAM-functionalized hydrogel effectively suppresses inflammation (Fig. 5H and L). Furthermore, immunofluorescence staining for CD206 demonstrated low CD206 signal intensity in both the control and UVB groups, with a modest increase observed in the HA group. In contrast, the HA/DAM group exhibited the most significant increase in CD206 intensity, indicating that DAM-functionalized hydrogel promotes M2 macrophage polarization, thus fostering an immune microenvironment conducive to skin regeneration and repair (Fig. 5I and M).

In this study, DAM-functionalized hydrogel exhibited substantially enhanced therapeutic efficacy over plain HA hydrogel, likely due to the synergistic interplay between DAM’s immunomodulatory properties and HA’s delivery capabilities. The enzymatic degradation of HA in vivo enables the controlled, site-specific release of DAM components, promoting their effective diffusion into the dermal layer. As a decellularized matrix material, DAM preserves essential ECM components and bioactive factors, which collectively modulate macrophage polarization and facilitate ECM repair. In addition, anti-inflammatory cytokines secreted by M2 macrophages directly activate fibroblasts, stimulating collagen synthesis and concurrently inhibiting MMP expression. In contrast, plain HA primarily exerts its effects through physical filling and hydration, lacking the capacity to actively regulate the inflammatory microenvironment, thereby offering limited efficacy in addressing the chronic inflammatory state characteristic of photoaged skin [37,38]. Collectively, DAM-functionalized hydrogel promotes the phenotypic shift from M1 to M2 macrophages, effectively attenuating inflammatory responses and fostering skin tissue regeneration and repair.

DAM-functionalized hydrogel demonstrates notable potential for clinical translation, offering a promising strategy for skin rejuvenation. First, DAM is derived from adipose tissue, a highly abundant and ethically uncomplicated source, which facilitates its scalability and clinical application. Second, our experimental results revealed that DAM-functionalized hydrogel exhibited excellent biocompatibility, as it did not induce inflammation or adverse effects during its degradation in vivo. These findings align with previous studies highlighting the safety and biocompatibility of decellularized matrices in regenerative medicine [39]. Furthermore, DAM-functionalized hydrogel demonstrated the ability to modulate the immune microenvironment, promoting a proregenerative milieu characterized by increased collagen density and dermal thickness. This is consistent with the well-documented role of M2 macrophage polarization in tissue repair and antiaging [31]. Collectively, these properties highlight the promise of DAM-functionalized hydrogels as innovative therapeutic strategies for skin rejuvenation.

Nevertheless, the current research has certain limitations that require further investigation. First, the limited number of mice used in the experiment may restrict the broader applicability of the results. Subsequent research should incorporate expanded sample sizes to confirm the consistency and reliability of the findings. Second, while short-term experiments demonstrated favorable outcomes, the long-term stability and efficacy of DAM-functionalized hydrogel remain to be confirmed through extended follow-up studies. Third, the mechanisms by which DAM modulates the immune microenvironment are likely multifaceted, involving various cell types and signaling pathways. A more comprehensive investigation is required to thoroughly clarify these mechanisms and enhance the therapeutic efficacy of DAM-functionalized hydrogel.

Future research will focus on optimizing the formulation of DAM-functionalized hydrogel, including enhancing stability, improving bioactivity, and modulating degradation kinetics to enhance its functional performance. Preclinical studies will involve expanding sample sizes and systematically evaluating its therapeutic efficacy and safety in antiaging and tissue repair. Further investigations will explore its potential in addressing complex dermatological conditions, such as chronic wounds and scar formation, to broaden its therapeutic applications. By addressing these scientific and technical challenges, DAM-functionalized hydrogel may contribute novel insights and tools for advancing innovative therapies in regenerative medicine and dermatology.

## Conclusion

This study identifies chronic inflammation and ECM metabolic dysregulation as key drivers of photoaging, validated in UVB-induced mouse models. In vitro, DAM, prepared via an enzyme-free decellularization method, attenuated inflammation by promoting M1 to M2 macrophage polarization. Moreover, DAM-stimulated macrophage CM restored UVB-impaired fibroblast function, reducing ROS levels, enhancing cell migration, and improving ECM homeostasis. In vivo, DAM-functionalized HA hydrogel increased dermal thickness and collagen density, suppressed inflammation, and enhanced M2 macrophage infiltration. These findings highlight DAM as a promising biomaterial with dual immunomodulatory and repair functions for photoaging treatment, offering new insights into ECM-immune interactions in antiaging strategies. Future studies should optimize DAM production scalability and evaluate its long-term safety and efficacy.

## Materials and Methods

### Establishment of the skin-photoaging model

All animal experiments were approved by the Laboratory Animal Ethics Committee in Ninth People’s Hospital Affiliated to Shanghai Jiao Tong University School of Medicine (approval no: SH9H-2023-A872-1). The C57BL/6 mice (10 to 12 weeks of age) were chosen to develop skin-photoaging models, following a well-established protocol [27]. Briefly, the animals were anesthetized, and their dorsal skin was shaved. A UVB lamp (311 nm) was then placed 30 cm above the dorsal side. The modeling period lasted for 8 weeks, during which the irradiation intensity was gradually increased according to the minimal erythema dose (MED). Specifically, the irradiation dose was set at 60 mJ/cm^2^ (1 MED) during the initial 2 weeks, increased to 120 mJ/cm^2^ (2 MED) in the 3rd week, raised to 180 mJ/cm^2^ (3 MED) in the 4th week, and maintained at 240 mJ/cm^2^ (4 MED) from the 5th to the 8th week. Wrinkle formation was monitored by dorsal skin photography and quantified at the end point using an established ImageJ-based method [27]. The standardized protocol involved threshold segmentation to isolate wrinkles from normal skin textures, followed by automated particle analysis to determine wrinkle count and morphological parameters. After that, the skin tissues of the photoaging mice were collected. One part was preserved in 4% paraformaldehyde (PFA; MM1504, Maokang Biotechnology Co. Ltd., Shanghai, China), while the other part was rapidly frozen in liquid nitrogen and stored at −80 °C.

### RNA isolation and qRT-PCR assay

The skin tissues of the photoaging mice were harvested, and the supernatants were collected using TRIzol reagent after thorough grinding. RNA was extracted following treatment with chloroform, isopropanol, and 75% ethanol solution and then reverse-transcribed into cDNA. Subsequently, qRT-PCR experiment was conducted using the LightCycler 96 PCR System. The sequences of primers for the target genes were provided in Table, with *Gapdh* acting as the reference gene for normalization.

**Table.** Primer sequences used in the present study

Genes	Forward sequences (5′–3′)	Reverse sequences (5′–3′)
*m-Col1a1*	GAGAGGTGAACAAGGTCCCG	AAACCTCTCTCGCCTCTTGC
*m-Col3a1*	GTGGCAATGTAAAGAAGTCTCTGAAG	GGGTGCGATATCTATGATGGGTAG
*m-Timp1*	CAGTGTTTCCCTGTTTATCTATCCC	GCAAAGTGACGGCTCTGGTAG
*m-Tgfb1*	CGTCACTGGAGTTGTACGGC	GTTTGGGGCTGATCCCGTTGA
*m-Il1b*	AGTTGACGGACCCCAAA	TCTTGTTGATGTGCTGCTG
*m-Il6*	AGCCCACCAAGAACGATAG	GGTTGTCACCAGCATCAGT
*m-Tnf*	CGCTGAGGTCAATCTGC	GGCTGGGTAGAGAATGGA
*m-Arg1*	ACGGTCTGTGGGGAAAG	TCAGGGGAGTGTTGATGTC
*m-Nos2*	GAGCGAGTTGTGGATTGTC	CCAGGAAGTAGGTGAGGG
*m-Il4*	GGTCTCAACCCCCAGCTAGT	GCCGATGATCTCTCTCAAGTGAT
*m-Il10*	AGGGTTACTTGGGTTGCC	GGGTCTTCAGCTTCTCACC
*m-Gapdh*	CCTCGTCCCGTAGACAAAATG	TGAGGTCAATGAAGGGGTCGT
*h-IL1B*	ATCAGCACCTCTCAAGCAG	AGTCCACATTCAGCACAGG
*h-IL6*	TGGCAGAAAACAACCTGA	GGCAAGTCTCCTCATTGAA
*h-COL1A1*	CCCCTGGAAAGAATGGAGATG	AGCTGTTCCGGGCAATCCT
*h-COL3A1*	CCCCGTATTATGGAGATGAACC	CCATCAGGACTAATGAGGCTTTC
*h-MMP1*	GGACCATGCCATTGAGAAAGC	TTGTCCCGATGATCTCCCCT
*h-MMP3*	CAATCCTACTGTTGCTGTGCG	GGACCACTGTCCTTTCTCCTAAC
*h-TIMP1*	CAATTCCGACCTCGTCATCAG	GGTTGTGGGACCTGTGGAAGTA
*h-GAPDH*	GGAAGCTTGTCATCAATGGAAATC	TGATGACCCTTTTGGCTCCC

m, primers for mice; h, primers for humans.

### RNA sequencing assay

The skin tissues of the photoaging mice were harvested, and the RNA sequencing (RNA-seq) analysis was carried out with the support of BGI Genomics Co. Ltd. Subsequent data analysis was performed using the Xiantao Academic website.

### Preparation of DAM

DAM was prepared from discarded human adipose tissue, with approval from the Ethics Committee of the Ninth People’s Hospital Affiliated to Shanghai Jiao Tong University School of Medicine (approval no: SH9H-2020-T138-2). The procedure was adapted from previously established protocols [28]. Briefly, the adipose tissues were first subjected to 3 freeze-thaw cycles, followed by mechanical emulsification using a 1.2-mm converter. Subsequently, the tissues were immersed in 0.5 and 1.0 M hypertonic salt solutions for 4 h each, followed by overnight incubation in deionized water. The samples were then exposed to 1% Triton X-100 solution for 48 h, followed by immersion in isopropanol solution for 8 h to remove lipids. Finally, the samples were washed 3 times with deionized water and 75% ethanol solution, respectively, and freeze-dried using a vacuum freeze dryer. To evaluate the biological effects of DAM, the DAM was further digested with pepsin (1 mg/ml) under constant stirring for 24 h, and the DAM solution was obtained and used in subsequent experiments.

### Characterization of DAM

The surface structure of DAM was examined with a stereoscope and SEM. To evaluate the decellularization efficacy, the DAM was processed through dehydration, embedding, and sectioning into paraffin blocks, followed by H&E staining in accordance with the kit protocols (G1120, Solarbio Science & Technology Co. Ltd). The residual DNA within the DAM was isolated using the DNA Extraction Kit (TIANGEN, China) and quantified using a spectrophotometer (Implen, Germany). The collagen and GAG contents were assessed using the Sircol Soluble Collagen Assay (Biocolor, UK) and the Blyscan GAG Assay (Biocolor, UK), respectively.

### Cell culture and cell proliferation

The RAW264.7 cells were obtained from the American Type Culture Collection and maintained in Dulbecco’s modified Eagle’s medium (DMEM; Gibco), supplemented with 10% fetal bovine serum (FBS; OriCell FBSSR-01021-500, Cyagen) and 1% penicillin–streptomycin (PS; PB180120, Procell). The cells were cultured in 37 °C incubators with 5% CO_2_. To evaluate the impact of DAM on cell proliferation, the RAW264.7 cells were seeded in the 96-well plates. After 24-h culture, the medium was changed to DAM solutions at various concentrations (0.125, 0.25, 0.5, and 1.0 mg/ml), and the medium was refreshed every 2 d. Following incubation periods of 1, 3, and 5 d, cell proliferation was evaluated using the CCK-8 method (JDB0101, BiOligo Biotechnology, Shanghai).

### Immunofluorescence staining

To evaluate the impact of DAM on the polarization of RAW264.7 macrophages, the cells were seeded in 24-well plates. After overnight culture, LPS solution (1 μg/ml) was initially added to simulate an inflammatory cellular state. After 24 h, DAM solutions at different concentrations were introduced. After 3 d of coculture, the cells underwent fixation, permeabilization, and blocking, followed by overnight incubation with the primary antibody at 4 °C and a 1-h treatment with the secondary antibody at 37 °C. Finally, the cells were stained with 4′,6-diamidino-2-phenylindole (DAPI) solution for 5 min and visualized under an inverted fluorescent microscope (Leica, Germany).

### HDF harvest

HDFs were derived from human foreskin specimens following a well-established methodology [36], with approval from the Ethics Committee of the Ninth People’s Hospital Affiliated to Shanghai Jiao Tong University School of Medicine (approval no: SH9H-2020-T138-2). Briefly, the foreskin tissues were rinsed with phosphate-buffered saline (PBS) solution supplemented with 5% PS–amphotericin B and cut into tissue blocks. The tissues were then placed in a culture dish and incubated with DMEM containing 5% Dispase II solution at 4 °C overnight. Subsequently, the dermal portion was collected and digested with 0.1% collagenase type I solution in a 37 °C shaker for 2 h. The digestion was stopped by adding an equal volume of DMEM. The mixture was filtered through a 70-μm cell strainer and centrifuged at 1,000 rpm for 5 min. The cell pellet was collected and cultured in complete DMEM. When cell confluency reached approximately 80% to 90%, the cells were passaged and used for subsequent experiments. For long-term storage, primary cells were resuspended in serum-free cell store freezing (catalog no. 40151ES50; Yeasen, Shanghai, China), preserved in cryogenic tube (607401, NEST Biotechnology), and stored in liquid nitrogen.

### Construction of the cell-photoaging model

The construction of the photoaging HDF model followed previously established methods [40]. The HDFs were plated in the culture dish, and upon achieving 40% confluency, the medium was discarded and substituted with 1 ml of PBS. A UVB lamp was placed 30 cm above the dish, and the cells received UVB irradiation of 50 mJ/cm^2^ daily for 3 d to induce photoaging.

### Preparation of macrophage CM

The macrophage CM was prepared to evaluate the modulation effect of DAM-induced RAW264.7 cells on the HDFs. Briefly, the RAW264.7 macrophages were plated in 6-well culture dishes. After induction with LPS for 24 h and DAM for an additional 3 d, the culture medium was refreshed. After 24 h, the cell supernatant was collected, centrifuged, and mixed with DMEM at a 1:2 ratio to obtain the CM.

### Intracellular ROS evaluation

The HDFs were plated in 24-well dishes and incubated overnight. Afterward, the medium was replaced with various CMs and cultured for 3 d. The medium was subsequently replaced with DCFH-DA working solution (KGA7501-1000, KeyGen Biotech), and the HDFs were maintained at 37 °C for 20 min. After that, the cells were examined under a fluorescence microscope, and the analysis was conducted using ImageJ software.

### Wound healing assay

The HDFs were seeded in 24-well plates and cultured with different CMs for 3 d. Upon achieving 90% to 100% confluency, a linear scratch was made across the well surface with a 200-μl sterile pipette tip. The cells were subsequently maintained in DMEM (2% FBS and 1% PS) for 24 h. Following this, the cells were fixed, examined, and photographed. Analysis was carried out using ImageJ software.

### Transwell migration assay

The HDFs were plated in 24-well plates and incubated with different CMs over 3 d. Subsequently, the cells were detached and suspended in DMEM without FBS. Then, the cell suspensions (200 μl) were placed in the upper compartment of the Transwell system, while 600 μl of complete culture medium was introduced into the lower chamber. After culturing for 24 h, the HDFs were fixed, stained by crystal violet solution, and imaged. Cell numbers were quantified using ImageJ software.

### Injectability

To evaluate the in vivo effects of DAM, commercial HA filler (Haiwei, Shanghai Haohai Biological Technology Co. Ltd., Shanghai, China) was thoroughly mixed with DAM using a 3-way connector. DAM was prepared as a solution of 10 mg/ml and mixed with HA in a volume ratio of 4:7 (DAM:HA). The injectability of the prepared HA/DAM hydrogel was evaluated by established methods [16]. Briefly, the DAM-HA mixture was filled into a 1-ml injector equipped with a 27-gauge needle. The injection pressure was assessed with a mechanical tester (Henyi, Shanghai, China) at a rate of 30 mm/min.

### FTIR spectroscopy analysis

FTIR analysis was conducted to characterize the chemical compositions of DAM, HA, and HA/DAM using an FTIR spectrometer (Great 20, Zhongke Ruijie Technology Co., China). Before analysis, the DAM solution, HA gel, and HA/DAM composite gel were lyophilized to obtain homogeneous solid samples. After background correction, the freeze-dried samples were placed on the attenuated total reflection (ATR) crystal, and spectra were recorded in the range of 4,000 to 400 cm^−1^. Characteristic chemical bonds and functional groups were identified on the basis of their respective absorption peaks.

### In vivo animal study

The established photoaged mouse models were used for the experiments (*n* = 5). The mice were anesthetized, and the back skin was depilated. HA and HA/DAM were administered subcutaneously into the bilateral back areas of the animals, with 200 μl of hydrogel administered per side. Photographs of the injection sites were taken weekly. To quantify hydrogel degradation kinetics, the residual gel volume was measured weekly. The volume (*V*) was calculated according to the established ellipsoid formula: *V* = (longest diameter) × (shortest diameter)^2^ × 0.52 [16]. Four weeks after injection, the skin tissues and major organs (heart, liver, spleen, lungs, and kidneys) were harvested and fixed in 4% PFA solution for further analysis.

### Histological staining

For histological staining, tissue samples were harvested and preserved in 4% PFA for 48 h. Following this, the samples were dehydrated, embedded, and sliced into thin sections. Before staining, the paraffin-embedded sections were cleared and rehydrated. For H&E and Masson’s trichrome staining, commercial kits were used (Masson’s trichrome, G1340, Solarbio Science & Technology Co. Ltd.), and the steps were carried out following the guidelines. For immunofluorescence and immunohistochemistry staining, the sections were treated with citrate-based antigen retrieval solution and underwent high-heat antigen retrieval. Following the inhibition of endogenous peroxidase activity and nonspecific interactions, the sections were treated with the respective primary antibodies (collagen I, collagen III, iNOS, and CD206) at 4 °C overnight, subsequently followed by exposure to secondary antibodies at 37 °C for 1 h. For immunohistochemistry staining, an additional step of 3,3′-diaminobenzidine staining and nuclear counterstaining was performed. Ultimately, the stained slides were examined under a microscope and photographed.

### Statistical analysis

Results are expressed as means ± SD. The quantitative experiments in this study were independently conducted a minimum of 3 times. Statistical evaluations were carried out using GraphPad Prism software. Differences between 2 groups were assessed using unpaired *t* tests, whereas differences among 3 or more groups were evaluated by one-way analysis of variance (ANOVA), followed by Tukey’s post hoc test for multiple comparisons. Statistical significance was determined as follows: ns, not significant; @, *, or #, *P* < 0.05; ** or ##, *P* < 0.01; *** or ###, *P <* 0.001; and **** or ####, *P <* 0.0001.

## Data Availability

Data will be made available on request.
